# Familial Patterns of Oral–Gut Dysbiosis and Systemic Markers in Periodontitis

**DOI:** 10.1111/jcpe.70047

**Published:** 2025-10-09

**Authors:** Hélvis E. S. Paz, Mabelle F. Monteiro, Camila S. Stolf, Cássia F. Araújo, Angelika Silbereisen, Mauro P. Santamaria, Nagihan Bostanci, Renato C. V. Casarin

**Affiliations:** ^1^ Division of Periodontics, Department of Prosthodontics and Periodontics, Faculdade de Odontologia de Piracicaba Universidade Estadual de Campinas Campinas Brazil; ^2^ Division of Oral Health and Periodontology, Department of Dental Medicine Karolinska Institutet Stockholm Sweden; ^3^ Division of Periodontics, Department of Diagnosis and Surgery Institute of Science and Technology – São Paulo State University São José dos Campos Brazil; ^4^ Division of Periodontology, Center of Oral Health Research College of Dentistry – University of Kentucky Lexington Kentucky USA; ^5^ Department of Periodontics and Endodontics, School of Dental Medicine University at Buffalo Buffalo New York USA

**Keywords:** gut microbiome, oral microbiome, parent–child relations, periodontitis, saliva

## Abstract

**Aim:**

To investigate whether periodontitis in parents is associated with differences in the faecal microbiome and systemic markers in both themselves and their children.

**Methods:**

Eighty participants were divided into four groups (*n* = 20): parents with periodontitis (PP); healthy parents (PC); and their respective children (CP, CC). Clinical periodontal parameters were recorded. Saliva and faecal bacterial DNA were analysed via 16S rRNA sequencing. Salivary lactoferrin, faecal calprotectin, gingival crevicular fluid cytokines (IFN‐γ, IL‐10, IL‐17, IL‐1β, IL‐4, TNF‐α) and urinary intestinal permeability markers (claudin‐2, ‐3, ‐4, haptoglobin) were quantified.

**Results:**

Parents with periodontitis showed distinct faecal microbiota profiles, which were mirrored in their children and significantly differed from controls. Claudin‐2 levels were elevated in both PP and CP groups (*p* < 0.05) and positively correlated with the oral dysbiosis index and the faecal Firmicutes/Bacteroidetes ratio.

**Conclusions:**

Parental periodontal health appears to influence the faecal microbiome and systemic markers in the offspring. These findings highlight a potential pathway for oral–gut microbial transmission and its relevance to systemic health, warranting further investigation.

## 1 Introduction

Periodontitis is an oral inflammatory disease caused by an imbalanced host's immune response to a dysbiotic polymicrobial biofilm. This condition progresses to affect the supporting periodontal tissues, leading to attachment loss, bone destruction and ultimately tooth loss (Lamont et al. 2018). The transition from periodontal health to disease is marked by oral microbiome alteration, characterised by successive changes in the structure and function of the subgingival microbial community, along with an increase in certain bacterial genera and species known as periodontopathogens (Hajishengallis and Lamont 2016). A key characteristic of stage III/IV and grade C periodontitis in young individuals is the familial clustering of cases and shared susceptibility factors. Studies have shown that children from those individuals have worse clinical conditions, higher levels of periodontopathogens and a similar pattern of subgingival colonisation compared to their parents, a phenomenon not seen in healthy families (Monteiro et al. 2015, 2021).

However, the effects of periodontitis extend far beyond the oral cavity, with mounting evidence over recent decades demonstrating that the oral–gut axis may serve as a critical pathway linking oral microbiome dysregulation and chronic inflammation to various systemic conditions, including inflammatory bowel disease, neurological diseases, colorectal cancer, diabetes, cardiovascular disease and rheumatoid arthritis, among others (Hajishengallis and Chavakis 2021). Although the biological mechanisms are not yet fully understood, preclinical studies suggest that increased oral pathogenicity can influence cellular trafficking between the oral cavity and the gastrointestinal tract, as well as trigger cross‐reactivity with oral antigens in the gut (Kitamoto et al. 2020; Bao et al. 2022). Some periodontopathogens have been shown to compromise the integrity of the intestinal epithelial barrier, down‐regulate the expression of tight junction proteins and exacerbate inflammation by promoting pro‐inflammatory cytokine expression in the gut environment (Arimatsu et al. 2014; Bao et al. 2022), providing evidence of how a dysbiotic oral microbiome may contribute to gut alterations. It is important to emphasise that gut permeability and inflammation can also be assessed through non‐invasive biomarkers to identify the risk of systemic disorders. These include members of the tight junction protein family, such as claudins and zonulin (and its related protein haptoglobin), as well as neutrophil‐derived proteins such as calprotectin and lactoferrin (Derikx et al. 2010).

Additionally, previous findings have shown that children of parents with periodontitis exhibit dysregulated oral colonisation from a very early age, with an altered microbial profile particularly evident during the mixed dentition phase (Reis et al. 2023). Considering that the shaping and maturation of the gut microbiome begin in infancy and continue throughout childhood (Derrien et al. 2019), we hypothesise that the microbiome of offspring from periodontitis patients with periodontitis may significantly influence the formers′ microbiota, potentially impacting their systemic health. Therefore, in this study, we aimed to characterise the salivary and faecal microbiomes, as well as intestinal permeability markers, of patients with periodontitis and their children, and to compare them with those of periodontally healthy families.

## 2 Methods

A detailed description of the methods employed in this study is contained in Data S1.

### 2.1 Ethics

This cross‐sectional study complied with all relevant ethical regulations, including the Declaration of Helsinki, and was approved by the Ethical Committee in Research of the Piracicaba Dental School (CAAE: 14667519.4.0000.5418). Signed informed consent and assent forms were obtained from parents and children before enrolment.

### 2.2 Study Design and Participants

This comparative cross‐sectional study was conducted in accordance with the STROBE guidelines for observational studies, with the primary outcome being the difference in faecal microbiome composition (beta‐diversity metric) between parents and children compared to controls.

This investigation adopted inclusion criteria from previous studies that reported a dysbiotic‐like oral microbiome pattern in the offspring of young parents with periodontitis and severe attachment loss (Monteiro et al. 2021; Reis et al. 2023), aiming to capture this specific patient profile.

Systemically healthy participants were recruited for this study and included in one of the following four groups:

#### 2.2.1 Parents Perio (PP)

Patients diagnosed with stage III/IV generalised, grade C (non‐smoking and normoglycaemic), periodontitis (Papapanou et al. 2018). Additionally, they were also < 35 years of age at the time of diagnosis and in good systemic health, with at least 8 teeth with probing depth (PPD) and clinical attachment level (CAL) > 5 mm (with at least 2 sites with PPD > 7 mm at diagnosis and at least 20 teeth in the oral cavity), attachment loss > 30% of teeth involved. The ratio between the percentage of the interproximal radiographic bone loss of the most affected site and the patient's age had to be > 1 (rapidly progressing disease).

#### 2.2.2 Children Perio (CP)

Systemically healthy children between 6 and 12 years of age, with father or mother included in the PP group, and permanent first molars and central incisors fully erupted.

#### 2.2.3 Parents Control (PC)

Adults presenting no history of clinical attachment loss due to periodontitis, gingival sulcus with PPD ≤ 3 mm, no radiographic proximal bone loss, at least 20 teeth in the oral cavity and good systemic health.

#### 2.2.4 Children Control (CC)

Systemically healthy children between 6 and 12 years of age, with father and mother periodontally healthy and one of them included in the PC group, and the permanent first molars and central incisors fully erupted.

Periodontal assessments were performed by a calibrated examiner. In the control group, only one parent per family was selected, but both the parents had to meet the criteria, with the choice of father or mother based on matching the gender of the adults with the periodontitis group.

### 2.3 Oral and Systemic Samples Collection

After the periodontal examination and data collection, patients were instructed to return within 1 week with fresh faecal and urine samples stored in a sterile container with ice which was provided at the previous appointment. At this point, unstimulated saliva and gingival crevicular fluid (GCF) were collected from each patient. All collected and received samples were immediately stored at −80°C until analysis.

### 2.4 Immunoassay Analysis

GCF was collected to assess the subgingival inflammatory cytokine profile. The levels of interferon (IFN)‐γ, interleukin (IL)‐10, IL‐17, IL‐1β, IL‐4 and tumour necrosis factor (TNF)‐α in GCF were determined using multiplexed fluorescent bead–based immunoassay (Luminex MAGPIX). Calprotectin levels were measured in the stool, and salivary lactoferrin levels were quantified using the same assay. Claudin‐2, ‐3, ‐4, and haptoglobin levels were also measured in urine samples via enzyme‐linked immunosorbent assay (ELISA).

### 2.5 Microbiome Analysis

Genomic DNA of saliva as well as of stool samples were processed, and the V3–V4 region of the 16S rRNA gene was sequenced and evaluated together. Bioinformatics analysis was performed using QIIME 22024.2 (Bolyen et al. 2019).

### 2.6 Microbial Dysbiosis Index

Two indices were used to assess potential microbiome dysregulation. The subgingival microbial dysbiosis index (SMDI), developed for periodontitis patients, was calculated using salivary sequencing data, and the taxonomy was assessed with the HOMD database (version 15.2) and CLR transformation (Chen et al. 2022). The normalised abundances of 26 key species (normobiotic and dysbiotic) were included. Group and age differences were analysed using two‐way repeated measures ANOVA.

For stool, the microbial dysregulation was calculated using a relative abundance of a count table collapsed at the phylum level, and the ratio between Firmicutes and Bacteroidetes (F/B), previously correlated with inflammatory diseases (Ley et al. 2006; Frank et al. 2007), was estimated. Differences between groups and age were tested using the Mann–Whitney and Wilcoxon tests, respectively.

### 2.7 Statistical Analysis

Data distribution was first evaluated for normality using the Shapiro–Wilk test. For all clinical analyses of parents and children, the unpaired Student's *t*‐test was used. For the evaluation of gender distribution, the Chi‐squared test was used. The levels of proteins between groups were analysed using either the Mann–Whitney test or the Student's *t*‐test. Multivariate analysis of systemic markers was performed using principal component analysis (PCA) and factor analysis. The systemic markers and dysbiosis indices were correlated with each other using Spearman's correlation. Statistical analysis of microbiome analysis is detailed in the relevant section. All analyses considered a significance level of 5%.

## 3 Results

### 3.1 Participant Demographics and Clinical Parameters

This study included 80 participants, with 20 individuals in each of the four groups who met the eligibility criteria. The number of participants to be recruited was determined based on previous studies (Monteiro et al. 2021; Reis et al. 2023), given the challenges of performing a sample size calculation for this study design. A post hoc test was conducted to confirm the power of our study, using beta‐diversity metrics for both saliva and stool as the primary variable. The power curve indicated that a sample size of 20 patients per group was sufficient to achieve an estimated power above 0.8 (Figure S2).

There were no significant differences in age or gender between the groups. Their clinical and demographic data are summarised in Table 1. As expected, parents with periodontitis exhibited worse periodontal clinical parameters than controls, while their children already presented significantly increased PPD and higher levels of BoP (*p* < 0.05). None of the recruited offspring exhibited clinical attachment loss, confirming the absence of periodontitis in children of both groups.

**TABLE 1:** Clinical and demographical data of the study participants.

	Parents		Children	
	Perio (*n* = 20)	Control (*n* = 20)	Perio (*n* = 20)	Control (*n* = 20)
Age (years ± SD)	35.5 ± 4.0	40.6 ± 5.4	8.6 ± 1.7	8.6 ± 1.4
Sex (*n* female)	14	14	9	9
PI (% + SD)	37.4 ± 0.2^a^	15.0 ± 0.1	34.2 ± 0.2	13.7 ± 0.1
PPD (mm ± SD)	3.4 ± 0.6^a^	2.1 ± 0.2	2.3 ± 0.4^a^	1.8 ± 0.1
CAL (mm ± SD)	3.6 ± 0.6^a^	0.1 ± 0.1	0.0 ± 0.0	0.0 ± 0.0
BoP (% ± SD)	55.6 ± 25.1^a^	16.2 ± 0.9	27.8 ± 0.1^a^	5.0 ± 0.0

Abbreviations: BoP, bleeding on probing; CAL, clinical attachment level; PI, plaque index; PPD, periodontal probing depth; SD, standard deviation.

^a^ Indicates the statistical difference between Perio and Control for each age group.

### 3.2 Oral Microbiome

**FIGURE 1:**
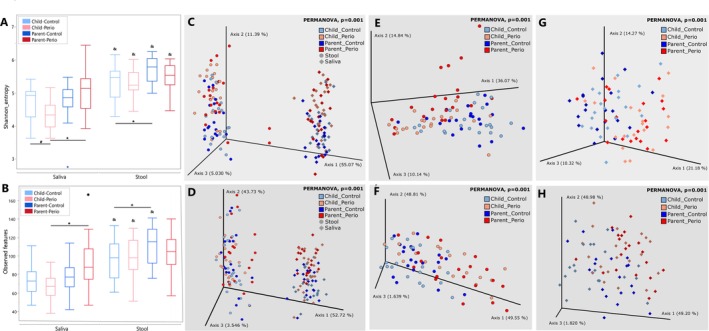
(A) Alpha‐diversity measured by the Shannon index. #Represents differences between Control and Perio Groups in each age, * shows differences between Parents and Children in each group and & shows differences between salivary and faecal microbiomes for each group (three‐way ANOVA for repeated measures, *p* < 0.05). (B) Alpha‐diversity measured by the observed features. *Represents differences between Parents and Children in each group and & shows differences between salivary and faecal microbiomes for each group (three‐way ANOVA for repeated measures, *p* < 0.05). (C) Principal coordinate analysis of weighted Unifrac distance in the saliva and stool samples. (D) Principal coordinate analysis of the Aitchison distance in the saliva and stool samples. (E) Principal coordinate analysis of the weighted Unifrac distance for saliva samples across different groups. (F) Principal coordinate analysis of the Aitchison distance for saliva samples across different groups. (G) Principal coordinate analysis of the weighted Unifrac distance for stool samples across different groups. (H) Principal coordinate analysis of the Aitchison distance for stool samples across different groups.

Microbiome analysis revealed distinct bacterial colonisation patterns in the oral and faecal microbiomes, emphasising the role of dynamic biogeography in composition. Faecal microbiomes exhibited higher alpha‐diversity than oral microbiomes for all groups, with the exception of the PP group regarding the number of observed features (Figure 1A,B). Children in the Perio group had lower salivary alpha‐diversity than their parents and children Control groups, while those in the CC group had lower stool alpha‐diversity than their parents (*p* < 0.05). Weighted Unifrac metrics showed saliva and faeces formed distinct clusters (Figure 1C,D, Table S1).

Salivary PCoA analysis (Figure 1E,F) revealed distinct clustering for the Perio and Control groups, with significant differences between PP and all other groups. Interestingly, control families showed higher microbial similarity between parents and children (*q* = 0.757), unlike Perio families, where children already displayed distinct salivary microbiota compared to CC group (*q* = 0.002).

**FIGURE 2:**
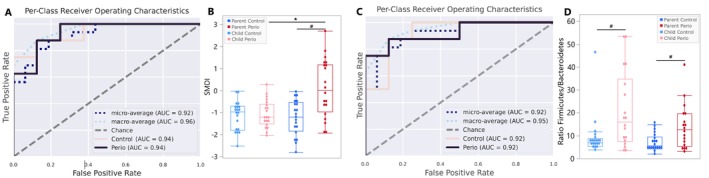
(A) Accuracy of the classification model for Perio and Control groups considering the salivary community at species level. The dashed line represents the reference line (AUC = 0.5), indicating random discrimination. The areas under the curve (AUC) for both the Perio and Control groups were 0.94, indicating the model's accuracy in classifying children and parents into the correct group based on microbial composition. (B) Subgingival microbial dysbiosis index (SMDI) in Parents and children in Perio and Control groups. #Represents differences between Control and Perio Groups, and * shows differences between Parents and Children in each group (two‐way ANOVA for repeated measures, *p* < 0.05). (C) Accuracy of the classification model for Perio and Control groups considering the gut community at genus level. The areas under the curve (AUC) for Perio and Control groups were 0.92, indicating the model's accuracy in classifying children and parents into the correct group based on microbial composition. (D) Firmicutes/Bacteroidetes (F/B) ratio in parents and children from Perio and Control group ^#^Represents differences between Control and Perio groups (Mann–Whitney test, *p* < 0.05), and * shows differences between parents and children in each group (Wilcoxon tests, *p* < 0.05).

Salivary microbiome differences between groups were confirmed using a classification model, dysbiosis index and differential abundance analysis. A random forest model based on parent–child similarities accurately classified samples into Perio and Control groups, as reported in the ROC curve (AUC = 0.92) (Figure 2A). Notably, children of Perio parents were classified as Perio based on species abundance, even without attachment loss, with key species linked to periodontitis (Figure S1). SMDI, a subgingival dysbiosis index, applied to the salivary microbiome, showed higher dysbiotic indices in Perio parents compared to Controls (*p* = 0.0005) and compared to their Perio children (*p* = 0.0008), although no significant difference was observed between groups in children (*p* > 0.05) (Figure 2B).

**FIGURE 3:**
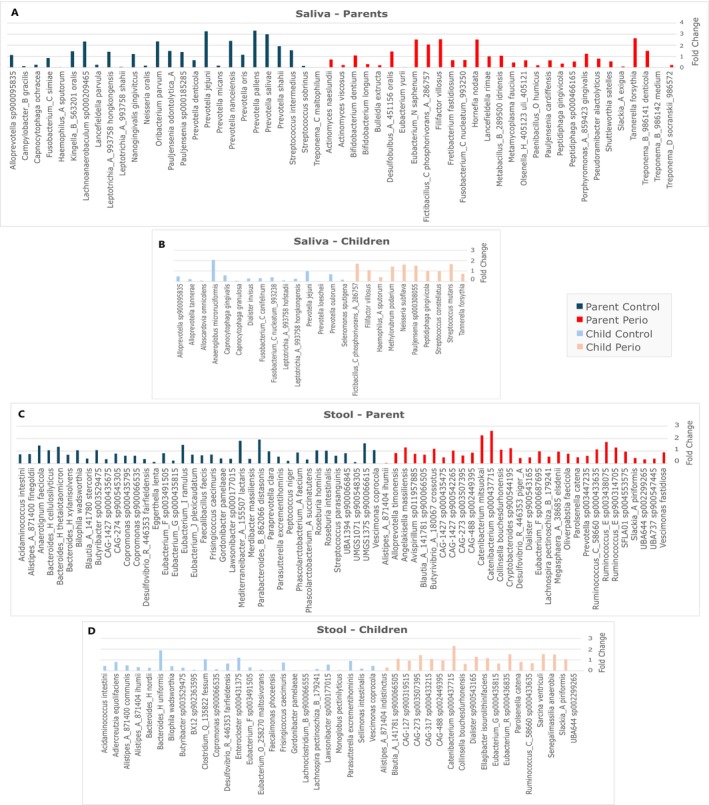
(A) Differentially abundant species between the Perio and Control groups in the salivary microbiome for the parents and (B) children included in the study. Only statistically significant differences between the groups, assessed by ANCOM‐BC, are included in the graph. The bars represent the fold‐change between the groups, and the colours represent the group in which the species is more abundant. (C) Differentially abundant species in faecal microbiome between the Perio and Control groups for the parents and (D) children included in the study. Only statistically significant differences between the groups (*p* < 0.05), assessed by ANCOM‐BC method, are included in the graphs. The bars represent the fold‐change between the groups, and the colours represent the group in which the species is more abundant.

Differential analysis of salivary species revealed increased biofilm pathogenicity in the Perio group compared to the Control (Figure 3A,B). Parents with periodontitis showed higher salivary levels of the red complex bacteria ( *Porphyromonas gingivalis* , *Tannerella forsythia* and *Treponema denticola* ) and other bacteria, including *Filifactor villosus* , *Fretibacterium fastidiosum*, *Fusobacterium_C_nucleatum_993250*, *Peptidiphaga gingivicola* and *Treponema_D socranskii_986572*. Notably, similar trends were observed in their children, with increased abundance of *F. villosus* , *P. gingivicola*, *T. forsythia* and *Streptococcus mutans* .

### 3.3 Faecal Microbiome

Differential microbiome analysis of groups revealed that the faecal microbiome beta‐diversity was influenced by the periodontal condition (Figure 1G,H). In addition to its impact on saliva, the diagnosis of periodontitis in parents was linked to a distinct pattern in the faecal colonisation of patients with periodontitis and their offspring (PP vs. PC, *q*‐value = 0.003; CP vs. CC, *q*‐value = 0.009), the children's gut community being highly similar to that of their parents (PP vs. CP, *q*‐value = 0.1464; PC vs. CC, *q*‐value = 0.155).

Regarding the faecal microbiome, a strong relationship with parental periodontal condition was seen in the children, as it was highly discriminative in beta‐diversity analyses and the classification model (ROC curve—AUC = 0.92) (Figure 2C), and in a dysbiosis index based on the phylum level (F/B ratio) (Figure 2D). Notably, there was a significantly elevated F/B ratio observed in children and parents belonging to the Perio group (PP vs. PC, *q*‐value = 0.0294; CP vs. CC, *q*‐value = 0.0054), with this difference being even more pronounced in children.

Furthermore, significant differences between the Perio and Control groups were also observed at the species level (Figure 3C,D). Among the common differences between groups for parents and children, the PP group showed reduced abundance of *Roseburia hominis* , *Roseburia intestinalis* and *Faecalibacillus faecis*. In contrast, they exhibited increased levels of *Butyrivibrio_a_180067 crossotus*, *Catenibacterium mitsuokai* , *Desulfovibrio_R_446353 piger_A*, *Prevotella sp003447235* and *Ruminococcus* species. Similarly, the comparison between children revealed a familial trend in microbial composition, with several species enriched in both Perio groups, including *Ruminococcus_C_58660 sp000433635*, *Dialister sp900543165* and *Catenibacterium sp000437715*, alongside a decreased abundance of others such as *Butyribacter sp003529475*.

### 3.4 Systemic Markers

**FIGURE 4:**
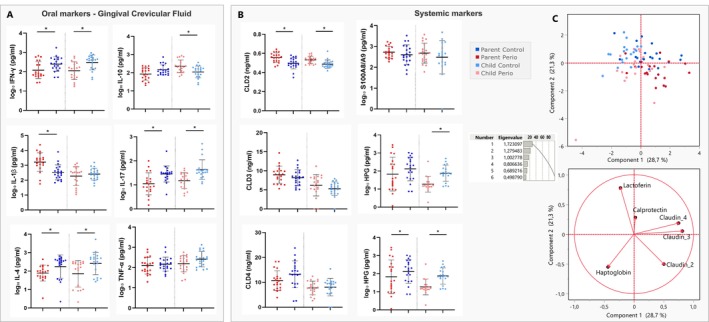
(A) Concentrations of inflammatory markers in gingival crevicular fluid (pg/mL). (B) Concentrations of permeability markers claudin‐2, ‐3, ‐4 (ng/mL) and haptoglobin (pg/mL) in urine, lactoferrin (pg/mL) in saliva and calprotectin (pg/mL) in stool. (C) Principal component analysis (PCA) of the systemic biomarkers. *Indicates statistical significance.

Figure 4A shows that Perio and Control groups presented significant differences in the cytokines' pattern in the GCF, showing a distinct profile for parents and children. Individuals in the PP group had significantly lower levels of IFN‐γ (*p* = 0.035), IL‐4 (*p* = 0.0086) and IL‐17 (*p* = 0.0027) and higher levels of IL‐1β (*p* = 0.0005) compared to those in the PP group, while those in the CP group had higher levels of IL‐10 (*p* = 0.0048) and lower levels of IFN‐γ (*p* = 0.0036), IL‐17 (*p* = 0.001) and IL‐4 (*p* = 0.0024) compared to those in the CC group.

Systemic markers related to intestinal permeability were assessed in saliva, urine and stool samples from families. Figure 4B shows the differences between groups, with the PP group members and their offspring showing higher levels of claudin‐2 (*p* < 0.001) and lower levels of haptoglobin (*p* = 0.0002) in urine. Furthermore, the PP and CP groups had significantly lower salivary lactoferrin levels (*p* = 0.0009 and *p* = 0.0047, respectively) than controls. No statistically significant differences were seen for other permeability markers (*p* > 0.05).

In the multivariate analysis of all systemic biomarkers, the systemic profiles of patients were determined using PCA (Figure 4C). The first and second components of the PCA represent 50% of the variability in the data. The second component separates the Perio and Control groups, while the first component tends to differentiate parents from children. In addition to PCA, a factor analysis was performed to correlate the systemic markers with the PCA components. Salivary lactoferrin was positively correlated with component 2, characterising the periodontal health status, while claudin 2 levels were associated with the Perio groups.

### 3.5 Integrative Analysis

**FIGURE 5:**
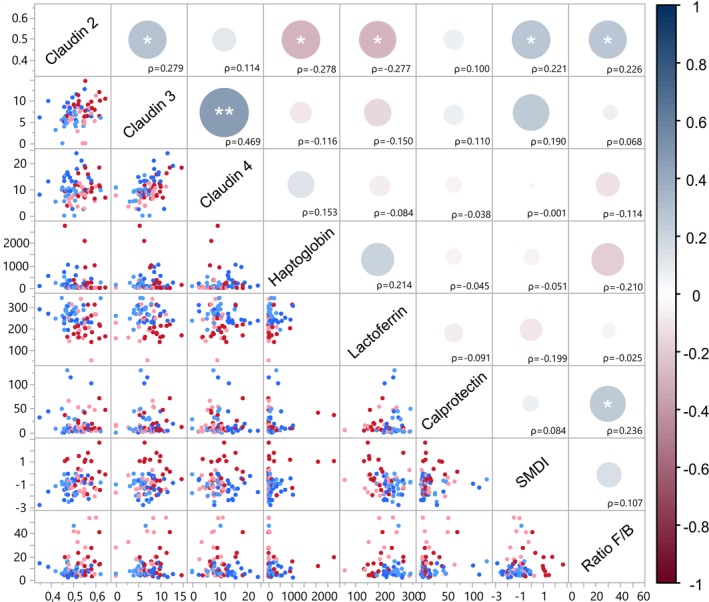
Spearman's correlation matrix between systemic markers and dysbiosis indexes. Levels of each systemic marker (calprotectin, lactoferrin, haptoglobin, claudin‐2, ‐3 and ‐4), the Oral Dysbiosis Index (SMDI) and the Firmicutes/Bacteroidetes (F/B) ratio were correlated, and significant associations are indicated (*p < 0.05; **p < 0.001). Colour denotes the direction of the correlation (blue for positive and red for negative), while the size of the circles represents the *r*‐values. The colour for the dots was defined by groups.

Interestingly, the close relationship between microbial dysregulation and systemic markers became more evident when we analysed all their interrelationships as independent variables. Figure 5 shows a positive association between SMDI and claudin‐2 (*ρ* = 0.221, *p* = 0.0485), as well as a negative correlation between claudin‐2 and salivary lactoferrin (*ρ* = *−*0.277, *p* = 0.0194) and haptoglobin (*ρ* = *−*0.278, *p* = 0.0145).

Additionally, our analysis revealed a significant correlation between the faecal microbiota F/B ratio and both faecal calprotectin (*ρ* = 0.236, *p* = 0.0449) and claudin‐2 (*ρ* = 0.226, *p* = 0.0495) levels. Additionally, a moderate positive correlation (*ρ* = 0.469, *p* < 0.0001) was found between claudin‐3 and claudin‐4 levels.

## 4 Discussion

Maintaining a healthy gut microbiota is essential for host homeostasis and innate immune defence. Both endogenous and exogenous factors are known to disrupt gut microbiota dynamics, particularly during the critical first 3 years of life (Derrien et al. 2019). This is especially important for children, who are in the process of establishing their microbial composition, a foundation for their immune defence system. Early disruptions can impact both recovery and resilience, making the altered microbiome a long‐term threat. By demonstrating the interconnectedness of the oral–gut relationship, our findings suggest that alterations in the parents' oral microbiome may influence the offspring's oral and faecal microbial colonisation. This study underscores the importance of examining the periodontal condition and associated oral microbiome as an influencing factor of the offspring's gut microbiome.

The acquisition and development of the gut microbial community are strongly influenced by family‐related factors. Genetics, lifestyle and dietary patterns, environmental exposures and co‐habitation are associated with shaping the gut microbiome composition (Brito et al. 2019; Ferretti et al. 2018; Valles‐Colomer et al. 2022). Similarly, mother‐to‐infant transmission and familial aggregation are the most accepted theories that might explain the high occurrence of periodontitis at early onset in members of the same family. Our findings support previous evidence that children from young parents with periodontitis already show a more pathogenic and distinct oral microbiome compared to those of periodontally healthy parents during the mixed dentition phase (Monteiro et al. 2021; Reis et al. 2023).

Recent research suggests the environment may influence oral and gut microbiomes more than genetics. Children's salivary microbiota showed higher strain‐sharing within households compared to gut microbiota (Valles‐Colomer et al. 2023). Children of periodontitis‐affected parents are more colonised by periodontopathogens in saliva, an easily transmissible fluid, indicating a significant parental impact on their microbiomes. Our findings further suggest that oral health may influence gut microbiota more strongly than life stage, as beta‐diversity analysis reveals distinct faecal microbiome similarities within parent–child pairs, differing significantly between Perio and Control groups. Previous studies have also shown age‐dependent variations in the gut microbiome, with school‐age adolescents having gut microbiomes that differ from those of adults and are more like one another (Hollister et al. 2015; Zhong et al. 2019). However, our results indicate that heredity, combined with oral microbial dysregulation, is a potential factor in shaping the gut community.

While intra‐familial similarity is evident, the faecal microbiome composition remains important. In this context, some microbes found in our analyses also appear to have an important impact on systemic health, as they were found significantly associated in studies evaluating systemic conditions. Genera such as *Bacteroides* and *Butyribacter*, which were decreased in the Perio groups for both parents and children, are linked to gut homeostasis, immune modulation, and beneficial effects on host health (Wexler and Goodman 2017; van den Bogert et al. 2014; Louis and Flint 2017). In both parents and children, the F/B ratio in Perio, a common marker of gut health, was altered, and previous studies have shown that this marker is an indicator of altered gut microbiome and systemic diseases (Yang et al. 2015; Rodiño‐Janeiro et al. 2018).

Altogether, the distinct faecal microbial community observed in parents and children from the Perio group appears to exhibit traits indicative of microbial dysregulation, which may explain the observed changes in immunological and permeability markers. Both Perio groups had elevated claudin‐2 levels in urine, indicating increased intestinal permeability, immune dysregulation and systemic inflammation (Prasad et al. 2005; Oami et al. 2024). Additionally, lower salivary lactoferrin levels in children from the Perio group could signal a threat to oral and systemic health. Lactoferrin plays a key role in antimicrobial defence and immune regulation, with its deficiency linked to periodontitis and heightened inflammatory responses (Fine 2015; Stolf et al. 2024; Liu et al. 2023).

The correlation between the oral dysbiotic index and elevated claudin‐2 and faecal calprotectin levels in the Perio group supports the oral–gut axis imbalance. High calprotectin is a marker of chronic gut inflammation, driving disease through cytokine activation and reactive oxygen species production (Sands 2015; Wiredu Ocansey et al. 2023). A study showed that transferring salivary microbes from periodontitis patients increased tight junction protein expression and altered inflammatory markers (Bao et al. 2022), consistent with our GCF findings. Collectively, we hypothesise that there may be an initial change in gut permeability, further evidenced by the increased F/B ratio, and that oral dysregulation could be an early indicator of future intestinal changes, and we must recognise the systemic impact of maintaining good oral health and consider the influence of familial conditions on children's microbiome development. This finding is particularly relevant, as *Bacteroidetes* members show a more distinct prevalence pattern in children than in healthy adults (Derrien et al. 2019), and children from parents with periodontitis already presented altered F/B ratio compared with children from parents without a periodontitis history.

Interestingly, higher GCF levels of IL‐10 were found only in children from the Perio group. Considering that these children presented significantly greater gingival inflammation but no attachment loss, IL‐10 may be produced as part of a successful attempt by the immune system to control ongoing inflammation. This contrasts with established periodontitis, in which the local IL‐10 response tends to be hyporesponsive (Stolf et al. 2023). In addition, the reduced levels of IFN‐γ may reflect a more destructive immune response and increased susceptibility to disease, a pattern previously observed in individuals with similar clinical characteristics (Monteiro et al. 2022). IFN‐γ plays a key role in infection control by promoting phagocytosis and inducing the production of inflammatory cytokines and molecules, and it has been suggested to exert a protective effect against the pathogenesis of periodontitis (Pan et al. 2019).

Certain limitations must be acknowledged. The relatively small sample size limits the generalisability of the findings. Also, the cross‐sectional design may not fully capture the dynamic nature of microbial colonisation. Additionally, the lack of dietary records limits the interpretation of gut microbiome composition, given the known impact of diet. Owing to the overall good health of participants and the inclusion of children, it was not feasible to collect blood‐based systemic markers commonly used in similar studies. Finally, the absence of retrospective follow‐up prevented assessment of early‐life exposures or gut vulnerability during the first 3 years of life.

## 5 Conclusion

In conclusion, this study underscores the influence of periodontal status on the oral–gut microbiome and systemic markers of intestinal permeability, which may have long‐term implications not only for systemic health but also for future generations, highlighting the importance of considering parental periodontal condition as a significant variable. Future analyses should explore the contribution of external exposures to this altered microbiome to determine whether its composition increases susceptibility to systemic diseases.

## Author Contributions

R.C.V.C. conceived and designed the study. H.E.S.P. performed sample collection. H.E.S.P., M.F.M., C.S.S., C.F.A. and M.P.S. coordinated and performed patient recruitment. H.E.S.P., C.S.S. and A.S. performed the experiments. M.F.M. designed and performed bioinformatics analysis. H.E.S.P. and M.F.M. generated data visualisation. H.E.S.P., M.F.M., N.B. and R.C.V.C. analysed the data. H.E.S.P. and R.C.V.C. drafted the manuscript. All authors critically revised and approved the manuscript.

## Ethics Statement

Approval for this study was obtained from the Ethics Committee for Research/National Commission for Ethics in Research, number: 14667519.4.0000.5418 (Piracicaba Dental School—State University of Campinas). Written informed consent was received from all subjects prior to participation.

## Conflicts of Interest

The authors declare no conflicts of interest.

## Supporting information


**Figure S1:** Top 30 most important features for the sample‐classifier model at the species level in saliva and at the genus level in stool.

**Figure S2:** Post hoc power analysis based on the beta‐diversity of the saliva and gut microbiome using Aitchison distance.

**Table S1:** PERMANOVA and pairwise test results among different groups.

**Data S1:** Supplementary Methods.

## Data Availability

The data that support the findings of this study are openly available in Sequence Read Archives (SRA) at https://www.ncbi.nlm.nih.gov/sra, reference number PRJNA1158855.
