# Supplementary material for: Familial Patterns of Oral–Gut Dysbiosis and Systemic Markers in Periodontitis
Source: J Clin Periodontol. 2025 Oct 9;53(2):222–31. doi: 10.1111/jcpe.70047 (PMC12803660; doi:10.1111/jcpe.70047)

Top 30 important features for sample-classifier model at species in saliva

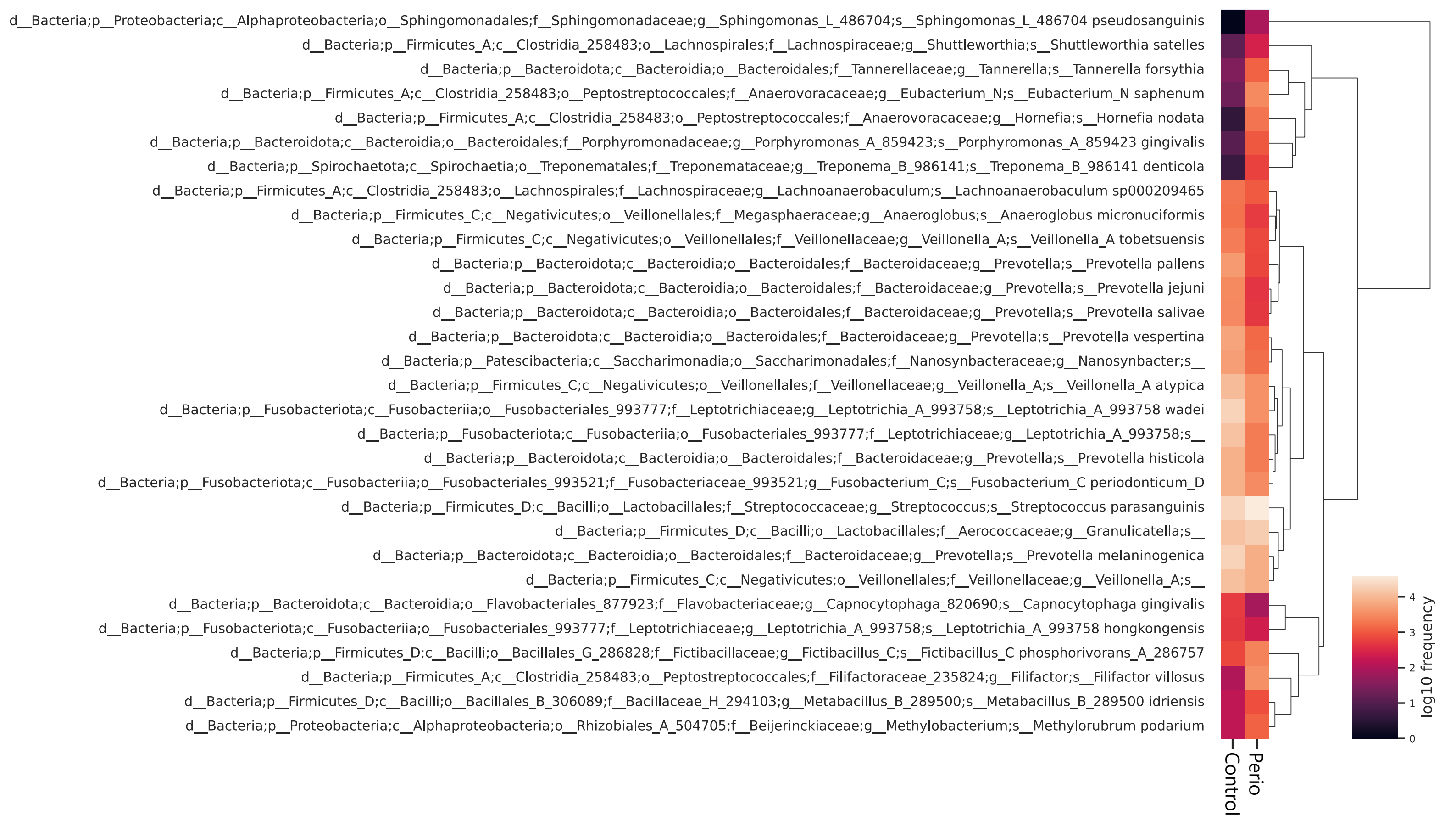

Top 30 important features for sample-classifier model at genus in stool

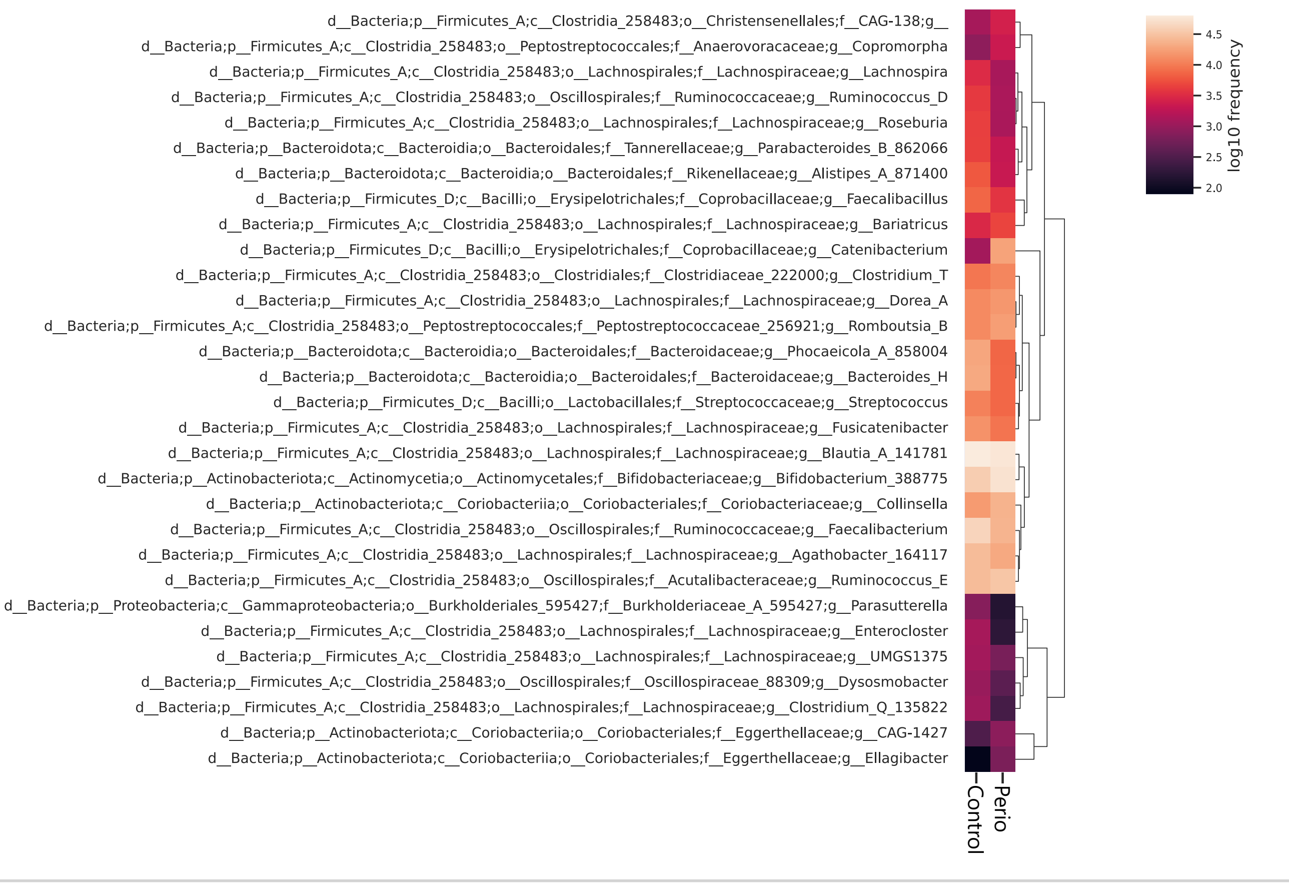

Supplement: Supplementary file 1 — Figure S1: Top 30 most important features for the sample‐classifier model at the species level in saliva and at the genus level in stool. [file JCPE-53-222-s001.pdf]
