# Supplementary figures and images for: Familial Patterns of Oral–Gut Dysbiosis and Systemic Markers in Periodontitis
Source: J Clin Periodontol. 2025 Oct 9;53(2):222–31. doi: 10.1111/jcpe.70047 (PMC12803660; doi:10.1111/jcpe.70047)

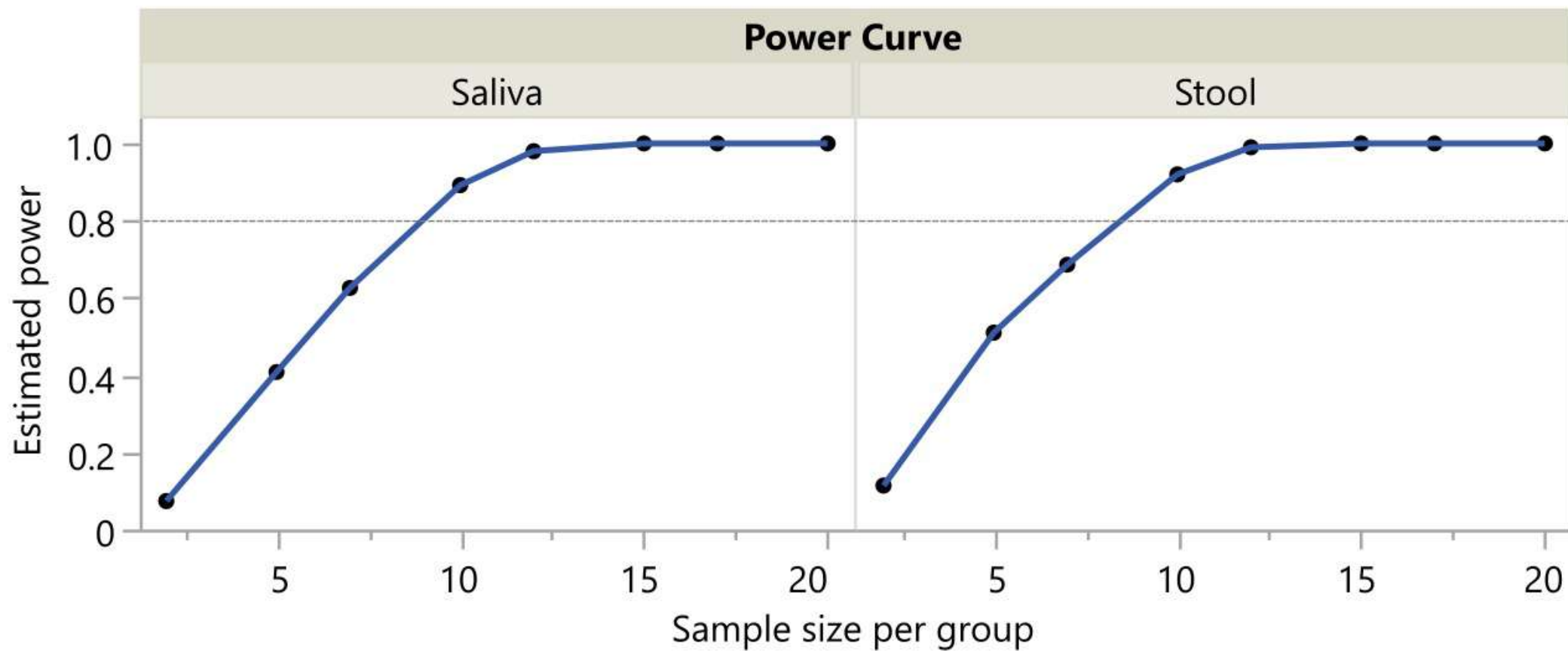

Supplement: Supplementary file 2 — Figure S2: Post hoc power analysis based on the beta‐diversity of the saliva and gut microbiome using Aitchison distance. [file JCPE-53-222-s003.pdf]
