# Supplementary material for: Familial Patterns of Oral–Gut Dysbiosis and Systemic Markers in Periodontitis
Source: J Clin Periodontol. 2025 Oct 9;53(2):222–31. doi: 10.1111/jcpe.70047 (PMC12803660; doi:10.1111/jcpe.70047)
Supplement: Supplementary file 4 — Data S1: Supplementary Methods. [file JCPE-53-222-s002.docx]

**Appendix**

**Title:** Familial Patterns of Oral-Gut Dysbiosis and Systemic Markers in Periodontitis

**Authors:** Hélvis Paz, Mabelle Monteiro, Camila Stolf, Cássia Araújo, Angelika Silbereisen, Mauro Santamaria, Nagihan Bostanci, Renato Casarin

**Appendix Methods**

**Study design and participants**

The study was conducted at Piracicaba Dental School, University of Campinas, Brazil, and was designed as a comparative cross-sectional study. The study protocol was developed following the STROBE guidelines for observational studies, and the primary outcome was the difference in fecal microbiome composition (beta-diversity metric) between parents and children compared to controls.

Patients were periodontally evaluated using a manual periodontal probe (UNC 15, HuFriedy, Chigaco, IL, USA), and the following full-mouth clinical parameters were collected: full-mouth plaque index (PI), periodontal probing depth (PPD), clinical attachment level (CAL), and bleeding on probing (BoP). The examination was conducted by a single and experienced examiner (HESP). In the control group, only one parent per family was selected, but the two parents had to meet the criteria, with the choice of father or mother based on matching the gender of the adults with the periodontitis group.

**Exclusion criteria**

Systemic exclusion criteria: the presence of reported systemic conditions (diabetes, obesity, cardiovascular disease, hepatitis, inflammatory bowel diseases, etc.) or use of antibiotics and chronic anti-inflammatory medication 6 months before the study, history of current or past smoking habits, pregnancy, and lactating.

Local exclusion criteria: Periodontal treatment including subgingival instrumentation in the 6 months prior to the study, use of orthodontic devices, alteration in the motor condition that modifies brushing habits. Sites with pulp and periapical pathologies, furcation lesions, enamel or dentin cracks, and defects were also excluded.

**Oral samples collection**

Unstimulated saliva (200μl) was collected by passive drooling from each patient, using a sterile 50mL conic tube and placed in labeled plastic tubes. All saliva samples were collected in the morning. Participants were asked to avoid eating, drinking and performing oral hygiene for at least one hour before the visit. Prior to the saliva collection, each participant rinsed their mouth with water and waited at least five minutes after rinsing to expectorate. The final volume was centrifuged for 5 min at 1500g at 4ºC to remove intact cells and debris. Then, the samples were sonicated for 10 min centrifuged at 10,000g for 5 min, and stored at -80ºC until microbiome analysis. Gingival crevicular fluid (GCF) was collected from two permanent first molars (one in the upper and one in the lower jaw) and two permanent incisors (one in the upper and one in the lower jaw), totaling four sites, using two sterile paper points per pocket/gingival sulci and then left in place for 15s. The papers were pooled and placed into sterile tubes containing 100μl of phosphate-buffered saline (PBS) with 0.05% Tween-20.

**Systemic samples collection**

After the periodontal examination and data collection, patients were instructed to return within one week with fresh fecal and urine samples, stored in sterile containers with ice packs provided at the previous appointment. The ice packs were used to prevent temperature increases during transportation, and patients were instructed to return to the research center as soon as possible after sample collection to avoid sample degradation. If immediate delivery was not possible, patients were instructed to store the sealed containers in their home freezer at –20°C until transport.

**Immunoassay analysis**

GCF collection was performed to evaluate the subgingival inflammatory cytokine profile. Levels of IFN-γ, IL-10, IL-17, IL-1β, IL-4, and TNF-α in GCF were determined using the MAGpix™ instrument and the Sensitivity Human Cytokine 06-plex kit (Millipore Corporation), according to the manufacturer’s recommendations. Samples were analyzed, and cytokine concentrations were estimated using the standard curve using a five-parameter polynomial equation using Xponent® software (Millipore Corporation). The mean concentration of each marker was calculated using the individual as a statistical unit and expressed as pg/ml.

To assess intestinal integrity and systemic inflammation, calprotectin levels were measured in feces using ELISA (Human S100A8/S100A9 Heterodimer, R&D Systems) and lactoferrin levels were measured in saliva using Luminex/MAGpix platform (Lactoferrin Human ProcartaPlex Simplex Kit), following the manufacturer’s instruction.

Claudin-2, -3, -4 (MyBioSource, CA, USA) and haptoglobin (R&D Systems) levels were quantified in urine samples through ELISA. Commercially available kits were used, following the manufacturer's instructions. Data were analyzed using the available standard curve, and a comparative analysis between groups was conducted.

Total protein from stool samples was isolated using RIPA lysis buffer with protease inhibitors (Jójárt et al. 2024), while for saliva and urine samples, the protein solution was obtained through successive centrifugations. All samples were stored at -80°C until analysis.

**Microbiome analysis**

Saliva samples were centrifuged at 8000 rpm for 1 minute, and the supernatant was removed. Bacterial DNA was incubated for 90-min with lysozyme (2 mg/ml) (Thermo Fisher Scientific) and then isolated using a Qiagen MiniAmp kit (Valencia, CA) according to the manufacturer's instructions. Fecal samples were processed by the RNeasy PowerMicrobiome kit (Qiagen, Hilden, Germany) according to an optimized protocol for DNA extraction (Falony et al. 2016).

Genomic DNA of saliva and stool samples were sequenced and evaluated together. A V3-V4 region of the 16S rRNA gene was amplified using primers 341F and 805R previously described (Klindworth et al. 2012). Then, PCR purification was performed according to the manufacturer's recommendation, using magnetic beads (AMPUre XP Bead, Beckman Agencourt) in the proportion of 0.8 spheres/PCR volume. After connecting the adapters, a new purification was performed by PCR using magnetic beads (AMPURE XP Bead, Beckman Agencourt) in the proportion of 1.12 beads/PCR volume. The normalization of the library was performed using the SequalPrep™ Normalization Plate kit (Applied Biosystems™), and the Pooled library was quantified using qPCR (KAPA Biosystems Library Quantification). Equimolar DNA concentrations were pooled and sequenced on the Illumina platform Miseq to produce 250pb end-paired sequences.

Bioinformatics analysis was performed using QIIME 2 2024.2 (Bolyen et al. 2019). The different sequence runs were processed separately and posteriorly merged for downstream analysis. Demultiplexed FASTQ files were obtained from the sequencing facility, and the q2-cutadapt (Martin 2011) was used to remove the primers. Denoising and quality control was performed with q2-DADA2 (Callahan et al. 2016). The taxonomy was assigned to ASVs using the q2-greengenes2 plugging and the Greengenes2 database (McDonald et al. 2023), and the analysis used the phylogenetic tree provided by Greengenes2 developers. The diversity metrics were estimated based on amplicon sequence variants (ASVs) using the q2-diversity core-metrics-phylogenetic pipeline. The alpha diversity (differences within samples) was estimated using the Shannon index and observed features, and the differences tested using three-way ANOVA. A beta diversity analysis was performed using the Weighted Unifrac and Aitchison distances and visualized with Principal Coordinate Analysis (PCoA). Differences in the distance matrix were tested using the PERMANOVA and the samples dispersion in was tested with PERMDISP. The subsequent analysis was performed using a table collapsed at the species level for saliva and at the genus level for stool samples. The core microbiome (taxa presented in at least 75% of samples in a group) was calculated using the core features from the q2-features-table and the visualization was performed using PhyloToAST (Dabdoub et al. 2016). The differential abundance between groups was assessed using ANCOM-BC (Lin et al., 2020) in RStudio (version 2024.04.0) and performed at the species level for both saliva and stool samples. This tool incorporated the FDR in the pipeline to correct for multiple comparisons, and the q-value was used to report the results. The plugging q2-sample-classifier (Bokulich et al. 2018) was used to build a classification model based on machine learning (random forest estimator), aiming to differentiate Perio from Control Groups in saliva and stool samples. The most important features to differentiate groups in the model were demonstrated in the Appendix figure, and the normalized feature abundance were represented in the heatmap using the q2-sample-classifier heatmap pipeline.

Post hoc power was calculated based on the primary outcome (beta diversity – Aitchison distance) and differences between groups assessed by PERMANOVA. Given the lack of standard analytical formulas for power estimation in this context, a simulation-based approach was applied directly to the dataset. Specifically, the distance matrix was repeatedly subsampled and PERMANOVA (adonis2, 999 permutations) was performed to empirically estimate the probability of detecting the observed effect at α = 0.05.

**REFERENCES**

Ainamo J,Bay I. 1975. Problems and proposals for recording gingivitis and plaque. *Int Dent J*. 25, 229–35.

Bokulich NA, Dillon MR, Bolyen E, Kaehler BD, Huttley GA, Caporaso JG. 2018. q2-sample-classifier: machine-learning tools for microbiome classification and regression. *J Open Res Softw*. 3(30):934.

Bolyen E. et al. 2019. Reproducible, interactive, scalable and extensible microbiome data science using QIIME 2. *Nat Biotechnol*. 37, 852–7.

Callahan BJ. et al. 2016. DADA2: High-resolution sample inference from Illumina amplicon data. *Nat Methods*. 13, 581–583.

Dabdoub SM. et al. 2016. PhyloToAST: Bioinformatics tools for species-level analysis and visualization of complex microbial datasets. *Sci Rep.* 6:29123.

Falony G. et al. 2016. Population-level analysis of gut microbiome variation. Science 352, 560–564.

Jójárt B, Resál T, Kata D, Molnár T, Bacsur P, Szabó V, Varga Á, Szántó KJ, Pallagi P, Földesi I et al. 2024. Plasminogen Activator Inhibitor 1 Is a Novel Faecal Biomarker for Monitoring Disease Activity and Therapeutic Response in Inflammatory Bowel Diseases. *J Crohns Colitis.* 2024 Mar 1;18(3):392-405.

Klindworth A. et al. 2012. Evaluation of general 16S ribosomal RNA gene PCR primers for classical and next-generation sequencing-based diversity studies. *Nucleic Acids Res.* 41, e1–e1.

Lin H, Peddada SD. 2020. Analysis of compositions of microbiomes with bias correction*. Nat Commun.* 11(1):3514.

Martin M. 2011. Cutadapt removes adapter sequences from high-throughput sequencing reads. *EMBnet J.* 17, 10.

McDonald D. et al. 2023. Greengenes2 unifies microbial data in a single reference tree. *Nat Biotechnol.* 42, 715–718.

Monteiro MF, Altabtbaei K, Kumar PS, Casati MZ, Ruiz KGS, Sallum EA, Nociti-Junior FH, Casarin RCV. 2021. Parents with periodontitis impact the subgingival colonization of their offspring. *Sci Rep.* 11(1):1357.

Mühlemann, HR, Son S. 1971. Gingival sulcus bleeding--a leading symptom in initial gingivitis. *Helv. Odontol. Acta*. 15,107–13.

Papapanou PN, Sanz M, Buduneli N, Dietrich T, Feres M, Fine DH, Flemmig TF, Garcia R, Giannobile WV, Graziani F et al. 2018. Periodontitis: Consensus report of workgroup 2 of the 2017 World Workshop on the Classification of Periodontal and Peri-Implant Diseases and Conditions. *J Clin Periodontol.* 45 Suppl 20:S162-S170.

Reis AA, Monteiro MF, Bonilha GM, Saraiva L, Araújo C, Santamaria MP, Casati MZ, Kumar P, Casarin RCV. 2023. Parents with periodontitis drive the early acquisition of dysbiotic microbiomes in their offspring. *J Clin Periodontol.* Jul;50(7):890-904.
